# Evaluation of Eye-Pain Severity between Dry-Eye Subtypes

**DOI:** 10.3390/diagnostics11020166

**Published:** 2021-01-25

**Authors:** Yamato Yoshikawa, Norihiko Yokoi, Hiroaki Kato, Rieko Sakai, Aoi Komuro, Yukiko Sonomura, Tsunehiko Ikeda, Chie Sotozono

**Affiliations:** 1 Department of Ophthalmology, Osaka Medical College, Takatsuki-City, Osaka 569-8686, Japan; yamt.0dec0@gmail.com (Y.Y.); tikeda@osaka-med.ac.jp (T.I.); 2 Department of Ophthalmology, Kyoto Prefectural University of Medicine, Kyoto 602-8566, Japan; hiro-kat@koto.kpu-m.ac.jp (H.K.); r-sakai@menicon.co.jp (R.S.); akomuro@koto.kpu-m.ac.jp (A.K.); yukky@ymail.plala.or.jp (Y.S.); csotozon@koto.kpu-m.ac.jp (C.S.)

**Keywords:** breakup pattern, dry eye, eye pain, eye fatigue, heavy eyelids, pain quantitative measuring device, questionnaire

## Abstract

The aim of this study was to assess eye pain between dry eye (DE) subtypes using questionnaires and the PainVision^®^ (Osachi) apparatus. This study involved 52 eyes of 52 DE patients with eye pain (43 females and 9 males; mean age: 64.2 ± 13.2 (mean ± SD) years) who were classified into three DE subtypes (aqueous deficient DE (ADDE); decreased wettability DE (DWDE); and increased evaporation DE (IEDE)) based on fluorescein breakup pattern. In all subjects, severity of eye pain was evaluated using PainVision^®^, the DE-symptom-questionnaire visual analog scale (DSQ-VAS), and the Short-Form McGill Pain Questionnaire 2 (SF-MPQ-2). The severity of eye pain was compared between the three DE subtypes. PainVision^®^ findings revealed greater severity of eye pain in ADDE and DWDE than in IEDE (*p* < 0.05, respectively), despite no difference being found in each questionnaire. A significant correlation was found between eye pain in DSQ-VAS and continuous pain, intermittent pain, neuropathic pain, and total pain in SF-MPQ-2 (R = 0.50, 0.49, 0.47, and 0.56, respectively) (all: *p* < 0.001). Greater severity of eye pain was found in ADDE and DWDE than in IEDE, and PainVision^®^ was found useful for the objective assessment of eye pain.

## 1. Introduction

According to the Asia Dry Eye Society (ADES) definition of dry eye (DE), it is defined as “a multifactorial disease characterized by unstable tear film causing a variety of symptoms and/or visual impairment, potentially accompanied by ocular surface damage” [1]. In the Tear Film and Ocular Surface Society (TFOS DEWSII) definition of DE, it is defined as “a multifactorial disease of the ocular surface characterized by a loss of homeostasis of the tear film, and accompanied by ocular symptoms, in which tear film instability and hyperosmolarity, ocular surface inflammation and damage, and neurosensory abnormalities play etiological roles” [2]. Thus, there are numerous similarities between those two definitions. However, the TFOS DEWSII definition places more attention on inflammation, hyperosmolarity, and neurosensory abnormalities. Moreover, in regard to classification subtypes, TFOS DEWS II classifies DE into two categories (i.e., aqueous deficient DE and evaporative DE). In contrast, ADES classifies DE into three categories (i.e., aqueous deficient DE (ADDE), decreased wettability DE (DWDE), and increased evaporation DE (IEDE)), which are differentially diagnosed through the classification of fluorescein breakup patterns (FBUPs) [3,4,5]. Moreover, of those subtypes, the latter two constitute short-breakup time (BUT) type DE (SBUTDE), which is characterized by shorter BUT with no or minimal involvement of decreased tear volume and ocular surface epithelial damage. These DE classifications are based on tear film dynamics, with each FBUP reflecting deficient components of the ocular surface: ADDE is caused by the deficiency of aqueous tears, DWDE is caused by the deficiency of membrane associated mucins, and IEDE is caused by the deficiency of the tear film lipid layer and secretory mucins. This originated as a new concept for DE from Asia and is different from that of TFOS [3,4,5,6]. This novel classification suggests the appropriate treatment of DE via the supplementation of each deficient component based on the FBUPs. It should be noted that there are some reports of new devices that can be focused on the evaluation of tear dynamics [7,8].

DE can present with various symptoms, so it is often pointed out that discrepancies in DE symptoms are sometimes noted between subjective and objective DE indications, thus subsequently producing a difficulty in correctly treating DE cases [9,10,11]. It should be noted that those discrepancies are especially marked in SBUTDE [12]. Among DE-related symptoms, special attention is placed on eye pain symptoms which are not only directly related to the patient’s quality of life, but are also sometimes extremely troublesome. It is important to note that in DE there is a crucial relationship between ocular surface inflammation and the ocular pain, in which the associated ocular pain may be caused through nerve changes triggered by DE-related inflammation with DE [13].

For the quantitative evaluation of eye pain, questionnaires such as those using visual analog scale (VAS) and the Short-Form McGill Pain Questionnaire 2 (SF-MPQ-2) are widely used. However, it has often been pointed out that the quantitative evaluation of DE-related symptoms using questionnaires has some limitations due to their subjective nature when used for evaluation. Questionnaires provide only a limited range of measurement and are restricted by fixed endpoints, that is, the maximum subjective value is set as the limit point, and the patients tend to evaluate the symptoms within that range, even if the stimulus exceeds the subjective value [14].

Recently, the PainVision^®^ (PS-2100; Osachi Co., Ltd., Nagano, Japan) apparatus, a new clinical device for the quantitative evaluation of the severity of pain, was developed, and it examines severity via a painless electronic stimulation. In the PainVision^®^ system, the underlying principle of measurement is to compare a unique electrical stimulation with the pain that the subject is actually experiencing. This device is currently being used mainly in the field of anesthesiology and in pain clinics in Japan [15,16,17,18,19,20,21]. To the best of our knowledge, there are no previous reports on the PainVision^®^ device being used to compare the severity of DE-related symptoms, including eye pain, etc., between the various DE subtypes. Thus, the purpose of this present study was to evaluate and compare the severity of DE-related symptoms, especially eye pain, between specific DE subtypes that were classified based on FBUPs, and to also analyze the relationship between questionnaires and the PainVision^®^ system when used as methods for the evaluation of DE-related symptoms.

## 2. Materials and Methods

The study protocols were approved by the Ethics Committee and the Institutional Review Board of Kyoto Prefectural University of Medicine, Kyoto, Japan (Approval No.: ERB-C-1233-3), and were carried out in accordance with the tenets set forth in the Declaration of Helsinki. Prior written informed consent was obtained from all subjects after a detailed explanation of the nature of the study and possible consequences associated with participation.

### 2.1. Subjects

This study involved 52 eyes of 52 DE patients with eye pain (43 females and 9 males; 29 right eyes and 23 left eyes; mean age: 64.2 ± 13.2 (mean ± SD) years, range: 33 to 87 years) seen at the Dry Eye Outpatient Clinic at the Kyoto Prefectural University of Medicine Hospital. In all patients, the eye with the more severe DE-related symptoms was selected. However, if the same severity in symptoms was observed bilaterally, the eye with a more definitive diagnosis as one of DE subtypes based on FBUPs was selected.

Exclusion criteria included any subjects who were using glaucoma eye drops, analgesics, or systemic steroids; who had diabetes mellitus; who had cardiac pacemakers, or the equivalent; who were pregnant or had planned pregnancy during the study period; who were within a 3-month range post ophthalmic surgery (including eyelid surgery, glaucoma surgery, and surgery for ocular surface disease); and who were contact lens users. In addition, all eyes with meibomian gland dysfunction (MGD) diagnosed based on the Japanese diagnostic criteria for MGD [22] were strictly excluded from the study due to the fact that MGD symptoms are not only derived from unstable tear film related to evaporative DE, but also from associated marginal blepharitis and accumulated lipids within the ducts of the meibomian glands. Furthermore, cases with DE accompanied by severe conjunctivochalasis, superior limbic keratoconjunctivitis, lid-wiper epitheliopathy, and filamentary keratitis, that is, when the subject’s symptoms could be explained solely based on those abnormalities, were also excluded from the analysis under the agreement of the evaluators (Y.Y., N.Y., H.K., A.K., and Y.S.). Since the purpose of this study was to specifically elucidate the relationship between eye pain and DE subtypes diagnosed based on FBUPs, all subjects were prohibited from using eye drops for at least 1 h prior to the examination in order to avoid any effect resulting from the instillation of the eye drops.

### 2.2. DE Subtype Classification

Each DE case was diagnosed based on the diagnostic criteria proposed by ADES and was subclassified into one of three subtypes based on FBUPs [3,4,5]. In regard to the FBUPs, DE cases showing an area break or line break FBUP were classified as ADDE, those showing a spot break or dimple break FBUP were classified as DWDE, and those showing a random break were classified as IEDE. In addition, the DE cases with a rapid expansion of line break were added as a type of DWDE [4,5,23]. Briefly, this type of FBUP shows a line break during the upward movement of fluorescein, however, the area of fluorescein breakup expands very rapidly in the manner of a horizontal direction when the eye is kept open, thus reflecting a decreased wettability of the corneal surface epithelium.

### 2.3. DE-Related Symptoms Evaluated by VAS-Based Questionnaires (DSQ-VAS)

The VAS is a tool used for the measurement of subjective symptoms that cannot be directly measured, and via this method, the DE-related symptom is evaluated by answering as “0” for no symptoms and as “100” for the most severe symptoms.

In this study, prior to the ocular surface examinations, the VAS was used to evaluate the DE-related symptoms in a questionnaire, that is, eye pain, dryness, blurred vision, sensitivity to light, eye fatigue, heavy eyelids, foreign body sensation, difficulty in opening the eye, redness, tearing, itchiness, and discharge. We define this questionnaire as DE-symptom-questionnaire visual analog scale (DSQ-VAS) in this study.

### 2.4. DE-Related Eye Pain Symptoms Evaluated by SF-MPQ-2

In this study, the SF-MPQ-2, a questionnaire that evaluates the pain experienced by each of the 22 pain descriptors using an 11-point numerical rating scale (0 = “none” to 10 = “worst possible”), was used to evaluate the severity of eye pain [24]. This questionnaire is primarily used in the field of anesthesiology and at specialized pain clinics, and is comprised of the following four pain subscales: (1) Continuous pain (throbbing pain, cramping pain, gnawing pain, aching pain, heavy pain, and tenderness), (2) Intermittent pain (shooting pain, stabbing pain, sharp pain, splitting pain, electric-shock pain, and piercing), (3) Neuropathic pain (hot-burning pain, cold-freezing pain, pain caused by light touch, itching, tingling or ‘pins and needles’, and numbness), and (4) Affective descriptors (tiring-exhausting, sickening, fearful, and punishing-cruel).

A total pain point is computed by the ratings provided by the subjects across all questions, while pain subscale points are derived from ratings to questions that comprise the respective scales.

### 2.5. Quantitative Evaluation of Eye Pain Using PainVision^®^

In this study, PainVision^®^ was used to objectively evaluate the severity of eye pain under natural blinks. As previously reported [15,16], the electrode part of the device that transmits electrical current is attached to the medial forearm. First, the current perception threshold (CPT), defined by the minimum electrical stimulation that could be sensed by the subject, was measured. Second, the pain equivalent current (PEC), defined by the electrical stimulation where the subject started to perceive the same strength as the current eye pain, was measured. Each measurement was performed three times, during which the reproducibility was confirmed, and the mean value of the three measurements was used as the measurement values. Through these measurements, pain degree (PD) was automatically calculated using the following equation:

PD = 100 × (PEC − CPT)/CPT(1)

It should be noted that each individual’s subjective sensation also plays an important role, as the device requires the subject to press a button in response to an electrical stimulus that is as strong as the subjective current eye pain.

### 2.6. Ocular Surface Examinations

The ocular surface examinations were performed in the following order. First, strict attention was placed on not increasing the subject’s aqueous tear volume, that is, after 2 drops of saline solution were put on a fluorescein test strip (Showa Yakuhin Kako Co., Ltd., Tokyo, Japan), the strip was vigorously shaken and the central portion of the top of the strip was gently placed on the central lower lid margin. After several natural blinks, the patient was then verbally instructed to gently close, and then briskly open, the eye. This verbal instruction was essential in order to clearly determine the starting point (time = 0 s) of the eye opening, as well as to confirm the reproducibility of the FBUP.

To determine the DE subtypes, FBUPs were evaluated simultaneously with the measurement of the fluorescein breakup time (FBUT), and scoring of the corneal and conjunctival epithelial damage was then performed.

The FBUT was measured by 1 evaluator (N.Y.) using a slit-lamp microscope with a cobalt blue filter for illumination and a blue-free filter for observation [25,26]. An electronic metronome was used for the measurement: the metronome sound beeped every 1 s in order to define the elapsed time from the start until the first appearance of a dark spot in the fluorescein-stained precorneal tear film when the eye was kept open. The FBUT was measured 3 times, and then averaged. Once the FBUTs were measured, the FBUPs were evaluated, and those that did not reproduce the same pattern three times were excluded. Fluorescein staining of the cornea and conjunctiva was observed using the blue-free filter system [25] and was scored depending on the severity of the staining. Staining of the cornea was scored in direct reference to the National Eye Institute scoring system [27], in which the cornea was divided into 5 portions and the staining was scored from 0 to 3 at each portion to calculate the total score on scales of 0–15 points. Staining of the conjunctiva was scored in direct reference to the van Bijsterveld scoring system [28], in which the staining was scored independently from 0 to 3 at the nasal and temporal bulbar conjunctiva in order to calculate the total score on scales of 0–6 points. As additional assessments of the ocular surface, ocular surface abnormalities often seen in association with DE, including conjunctivochalasis, superior limbic keratoconjunctivitis, lid-wiper epitheliopathy, and filamentary keratitis, were evaluated in order to exclude specific cases, as described above. Finally, as a test of tear secretion, the Schirmer 1 test was adopted using a standard Schirmer test strip (Showa Yakuhin Kako Co., Ltd., Tokyo, Japan) in which the wetted part of the strip from the top was measured under natural blinking 5 min after the insertion of the strip at the temporal one-third of the lid margin into the conjunctival sac.

### 2.7. Statistical Analysis

For comparison of the three DE subtypes, the Tukey-Kramer Honestly Significant Difference (HSD) test was used for parametric and continuous items, and the Steel-Dwass test was used for non-parametric or intermittent items. In both tests, a difference of *p* < 0.05 was considered statistically significant. The correlation coefficient was calculated using the Spearman’s rank correlation coefficient formula. A correlation coefficient of ≥0.4 and a *p*-value of <0.05 was considered statistically significant. For multivariate analysis, the relative risk was measured using the least squares method, and a *p*-value of <0.05 was considered statistically significant. JMP PRO version 15 (SAS Institute, Inc., Cary, NC, USA) statistics software was used for all statistical analyses.

## 3. Results

### 3.1. Patient Background for Each DE Subtype

The patient background of each DE subtype is shown in Table 1. In regard to age and sex, analysis of variance revealed no significant differences among the three subtypes. The average age ranged from 62.7 to 65.9 years, and females accounted for more than 70% of the subjects in all three groups. FBUT was significantly shorter in DWDE than in IEDE (*p* = 0.004). Similarly, FBUT was significantly shorter in ADDE than in IEDE (*p* = 0.004). The ADDE group had significantly greater corneal and conjunctival epithelial damage scores and significantly less Schirmer 1 test values than those in the other two groups (*p* < 0.0001 ~= 0.002).

### 3.2. Pain Subscale of DE-Related Symptoms Evaluated by DSQ-VAS and SF-MPQ-2

**Figure 1:**
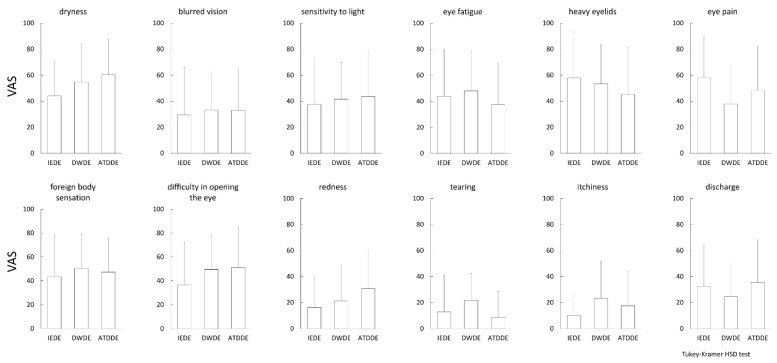
Dry eye (DE) related subjective symptoms evaluated by visual analog scale (VAS). The mean value of the VAS for each symptom is plotted for each DE subtype, and the standard deviation is shown as a vertical bar. The Tukey-Kramer HSD test was used for statistical analysis, and a *p*-value of <0.05 was considered statistically significant.

**Figure 2:**
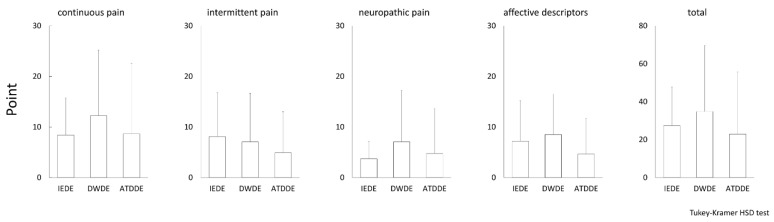
Eye pain evaluated by Short-Form McGill Pain Questionnaire 2. The mean value of the point for each pain subscale and total pain was plotted for each DE subtype, and the standard deviation is shown as a vertical bar. The Tukey-Kramer HSD test was used for statistical analysis, and a ***p***-value of <0.05 was considered statistically significant.

The comparative results of DSQ-VAS and SF-MPQ-2 among the three DE subtype groups are shown in Figure 1 and Figure 2, respectively. No significant differences were observed among the three groups in any of the symptom and pain subscales.

The results of the relationship between DE-related symptoms evaluated by DSQ-VAS and each pain subscale or total pain evaluated by SF-MPQ-2 are shown in Table 2. Corresponding scatterplots to Table 2 have been provided as a Supplementary Material Figure S1 significant correlation was found between eye pain evaluated by DSQ-VAS and continuous pain, intermittent pain, neuropathic pain, and total pain (R = 0.50, 0.49, 0.47, and 0.56, respectively) (all: *p* < 0.001) by SF-MPQ-2. Moreover, a significant correlation was found between eye fatigue/heavy eyelids evaluated by DSQ-VAS and continuous pain, affective descriptors, and total pain, respectively, done by SF-MPQ-2 (Eye fatigue: R = 0.47, 0.47, and 0.49, respectively; Heavy eyelids: R = 0.56, 0.51, and 0.55, respectively) (all: *p* < 0.001). No significant correlation was found between the other symptoms evaluated by DSQ-VAS and the pain subscales or total pain evaluated by SF-MPQ-2.

### 3.3. Comparison of PD Evaluated by PainVision^®^ among the DE Subtype Groups

**Figure 3:**
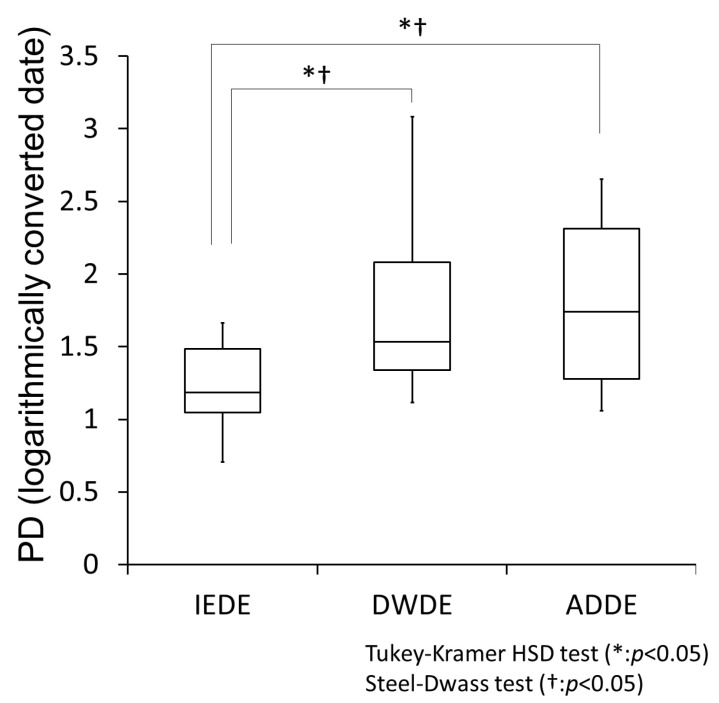
Box-and-whisker plot of minimum to maximum eye pain evaluated by PainVision^®^. The evaluated eye pain is expressed by a Pain Degree (PD) index, and the logarithmic conversion of PD is shown in this figure. The base of the logarithm is 10. The box contains the lower quartile, the median, and the upper quartile for each DE subtype, and the minimum and maximum values are indicated by vertical bars. The Steel-Dwass test and the Tukey-Kramer HSD test were used for statistical analysis, and a *p*-value of < 0.05 was considered statistically significant.

Psychological sensation is perceived increasingly in a logarithmic fashion, not directly proportional to the intensity of stimulus (Weber-Fechner law) [29]. It has previously been reported that this law is also applicable to the sensations of the ocular surfaces and body surface [30]. Therefore, it is necessary to take the logarithm when calculating the average. Figure 3 shows the results of PD measured by PainVision^®^ among the three DE subtype groups (the Y-axis data shows the logarithmically converted data of PD, with the number that is the base of the logarithm being 10).

It should be noted that both the Steel-Dwass test and the Tukey-Kramer HSD test were used. According to Weber-Fechner’s law, the Tukey-Kramer HSD test can be used when logarithmic transformation is performed. However, weak stimuli do not follow this law due to noise, so the Steel-Dwass test (a non-parametric test) is added. In both tests, PD was significantly greater in the ADDE and DWDE groups than in the IEDE group (*p* < 0.05, respectively).

### 3.4. Relationship between PD and Objective Findings

The relationship between PD and objective findings was evaluated. As shown in Table 3, there was no significant correlation between PD and any of the ocular surface examination findings. FBUT showed a tendency of negative correlation, but the result was not statistically significant.

Table 4 shows the multivariate analysis of the objective findings for PD by the least squares method. Since there were correlations between each ocular surface examination, four multivariate analyses with different variables were performed. In all analyses, the most significant effect on PD was found to be the DE subtype classification.

## 4. Discussion

Throughout the duration of this study, the evaluation of eye pain using PainVision^®^ showed that the severity of pain symptoms was significantly greater in the ADDE and DWDE subtypes than in the IEDE subtype. On the other hand, the evaluation of eye pain severity using the DSQ-VAS and SF-MPQ-2 questionnaires revealed that the severity of pain was not statistically significantly equal among all three DE subtypes.

In the comparison between DSQ-VAS and SF-MPQ-2, a significant correlation was found in various items. In particular, a significant correlation was found between eye pain evaluated by DSQ-VAS and continuous pain, intermittent pain, neuropathic pain, and total pain evaluated by SF-MPQ-2. Moreover, a significant correlation was found between eye fatigue/heavy eyelids evaluated by DSQ-VAS and continuous pain, affective descriptors, and total pain evaluated by SF-MPQ-2, respectively. Among the objective findings, the DE subtype classification had the strongest effect on eye pain measured by the PainVision^®^ quantitative pain analysis.

The findings of this study clarified the differences between questionnaires and PainVision^®^ in assessing DE-related eye pain. However, it should be noted that the findings in some reports from other medical fields have pointed out that there is no correlation between PD measured by PainVision^®^ and pain evaluated by questionnaires such as a visual analog scale (VAS) and SF-MPQ-2 [16,17]. The reason for the discrepancies between these two evaluation methods is probably the pain components that are evaluated. The International Association for the Study of Pain has defined pain as “an unpleasant sensory and emotional experience, associated with actual or potential tissue-damage or described in terms of such damage” [31]. Thus, pain is a subjective perceptual experience that has two defining properties: (1) a bodily sensory component and (2) an affective component. Questionnaires such as pain questionnaire using a VAS, or the SF-MPQ-2, are strongly influenced by emotional, social, and cultural components, not just the sensory components of pain. Moreover, patient questionnaires are easily influenced by personality variables. In other words, the subjectively determined “worst condition” may vary from patient to patient. On the other hand, PainVision^®^, which uses a heterogeneous sensation of electrical stimulation to evaluate pain, is less influenced by an affective component of pain, which is a difference that might have had an influence on the results in this current study.

DE-associated eye pain is a factor that significantly impairs the patient’s quality of life and is one of the challenges that must be overcome. However, pain symptoms tend to be unreliable, and dependent on the subjective evaluation by the patients, which is likely to result in an inappropriate treatment of DE. For example, there are several cases with IEDE in this study in which the results of strong pain were more obtained from the questionnaire than from PainVision^®^. In such cases, it is possible that psychological care may be a better avenue than direct DE treatment. Moreover, the results of this study suggest that subtype-specific treatments (i.e., from ADDE or DWDE to IEDE) may effectively treat eye pain symptoms. For example, for severe ADDE, the use of punctal plugs and eye drops that replenish aqueous tear fluid, and for DWDE, the use of eye drops such as diquafosol or rebamipide that improve the wettability of the ocular surface can be recommended [23,32,33].

Although the questionnaire format is influenced by various factors, it is beneficial from certain perspectives. For example, it is a format in which various pain and symptom variations are easily derived depending on the content of the questions, so it can provide new insights into pain. In this study, by comparing DSQ-VAS and SF-MPQ-2, our findings suggested that the eye pain associated with DE cases was complex with a variety of pains, including continuous, intermittent, and neuropathic pain. Eye fatigue and heavy eyelid were evaluated as pain in DE cases. Eye fatigue and heavy eyelid correlate with not only continuous pain but also affective descriptors, so they may not be fully evaluated by an evaluation via PainVision^®^. Eye fatigue and heavy eyelid may occur in association with asthenopia, as well as in association with DE. However, the underlying mechanism is not fully understood [34,35]. With that stated, it can be the basis for explaining discrepancies between objective signs of the ocular surface and eye pain in DE. In the future, the symptoms of eye fatigue and a heavy eyelid might be appropriately added to the evaluation of eye pain, and treatment approaches for both problems may possibly improve eye pain that has not previously been improved in the clinical setting.

The evaluation of eye pain using PainVision^®^ is objective and is useful for assessing the somatosensory component of eye pain. On the other hand, and although easily influenced by various factors, the questionnaire format is simple, thus making it useful for screening and for detecting cases suffering from non-sensory eye pain. Thus, when these two methods are combined, more variations of pain can be evaluated.

The findings in this study show that DE subtype classification based on FBUPs well-correlates with eye pain severity compared to other objective findings. Moreover, DE subtype classification is useful for selecting a specific treatment from the aspect of tear film stability. According to ADES, DE subtype classification is used to detect the insufficient components of the ocular surface comprising the tear film and the surface epithelium, and to supplement them for improved tear film stability (this concept is defined as “tear-film-oriented diagnosis” (TFOD) and “tear-film-oriented therapy” (TFOT) [4]. Accordingly, from this aspect, tear film stability can also be useful for evaluating eye pain. In a recent study by Dieckmann et al. [36], the authors suggested that the existence of a corneal nerve microneuroma is a sign of eye pain. In the future, the mechanism of eye pain may be further clarified by investigating the relationship between such new objective abnormalities and eye pain quantitatively evaluated by PainVision^®^.

One limitation of this present study was that it was the first to investigate the use of the PainVision^®^ system for DE, so it is necessary to confirm whether or not measurements being obtained under the natural blink process is the optimal method. For example, a type of test involving the eye being kept open may actually enhance eye pain in a manner that more closely mimics daily life, such as desk-related work. Moreover, the small sample size is another limitation of this present study, so further studies may be needed to reconfirm the results that were obtained.

Based on the results obtained from this present study, in DE cases presenting ocular pain, it is vital to confirm whether or not the present DE type is DWDE or ADDE via the observation of FBUPs. Further research is needed to confirm if appropriate treatment for those DE types will improve the associated ocular pain. It is also important to confirm whether or not the subjective ocular pain may be the result of general eye fatigue or a heavy eyelid.

## 5. Conclusions

The findings in this study revealed that when evaluating eye pain in DE cases, the PainVision^®^ device may be a useful method that can more effectively evaluate sensory components of pain than traditional questionnaire formats, as when diagnosed by FBUPs, PainVision^®^ demonstrated greater pain in ADDE and DWDE than in IEDE.

## Figures and Tables

**Table 1:** Demographic and Clinical Characteristics of Study Subjects.

	IEDE (*n* = 10)	DWDE (*n* = 22)	ADDE (*n* = 20)	*p* (IEDE vs. DWDE)	*p* (IEDE vs. ADDE)	*p* (DWDE vs. ADDE)
Demographic						
Age, years, mean (SD)	65.9 (15.9)	64.9 (12.2)	62.7 (13.4)	one way ANOVA *p* = 0.78		
Female, *n* (%)	7 (70.0%)	19 (86.4%)	17 (85.0%)	one way ANOVA *p* = 0.51		
Ocular Surface Evaluations, mean (SD)						
FBUT, s $	5.2 (2.5)	1.9 (1.7)	2.0 (1.7)	0.004	0.004	1.0
Schirmer 1 test, mm $	20.0 (12.1)	19.7 (11.3)	5.3 (5.8)	1.0	0.002	<0.0001
Corneal staining score (0–15) #	1.0 (1.2)	1.0 (1.4)	8.0 (3.3)	1.0	<0.0001	<0.0001
Conjunctival staining score (0–6) #	0.1 (0.3)	0.5 (1.0)	3.5 (2.2)	0.81	<0.0001	<0.0001

#: Tukey-Kramer HSD test; $: Steel-Dwass test; IEDE: increased evaporation dry eye; DWDE: decreased wettability dry eye; ADDE: aqueous deficient dry eye; FBUT: fluorescein breakup time. Underline is added when *p* value is less than 0.05.

**Table 2:** Correlation between subjective symptoms of dry eye evaluated by VAS and SF-MPQ-2, evaluated by points.

	SF-MPQ-2									
	Continuous Pain		Intermittent Pain		Neuropathic Pain		Affective Descriptors		Total Pain	
VAS	R	*p*	R	*p*	R	*p*	R	*p*	R	*p*
Dryness	0.15	0.30	0.09	0.50	0.09	0.51	0.16	0.27	0.13	0.35
Blurred vision	0.34	0.01	0.17	0.21	0.30	0.03	0.18	0.20	0.30	0.03
Sensitivity to light	0.22	0.11	0.32	0.02	0.19	0.17	0.14	0.32	0.26	0.06
Eye fatigue	0.47	0.0004	0.27	0.05	0.16	0.26	0.47	0.0004	0.49	0.0002
Heavy eyelids	0.56	<0.0001	0.33	0.01	0.19	0.17	0.51	0.0001	0.55	<0.0001
Eye pain	0.50	0.0001	0.49	0.0002	0.47	0.0003	0.25	0.07	0.56	<0.0001
Foreign body sensation	0.36	0.008	0.35	0.01	0.30	0.03	0.15	0.30	0.37	0.007
Difficulty in opening the eye	0.24	0.08	0.07	0.62	0.11	0.42	0.06	0.66	0.18	0.20
Redness	0.06	0.65	0.22	0.12	0.17	0.24	−0.05	0.70	0.11	0.44
Tearing	0.28	0.04	0.21	0.12	0.16	0.25	0.08	0.56	0.23	0.10
Itchiness	0.24	0.08	−0.01	0.94	0.13	0.35	0.10	0.47	0.14	0.32
Discharge	0.08	0.56	0.22	0.12	0.16	0.24	0.04	0.75	0.14	0.32

VAS: visual analog scale; SF-MPQ-2: Short-Form McGill Pain Questionnaire 2; R: Spearman’s rank correlation coefficient. Underline is added when the absolute value of R is 0.4 or more.

**Table 3:** Correlation between PD and Ocular Surface Evaluations.

	PD		FBUT		Schirmer 1 Test		Corneal Staining Score		Conjunctival Staining Score	
Ocular Surface Evaluations	R	*p*	R	*p*	R	*p*	R	*p*	R	*p*
PD			−0.24	0.089	−0.04	0.80	0.14	0.33	0.13	0.35
FBUT	−0.24	0.089			0.26	0.065	−0.44	0.001	−0.17	0.24
Schirmer 1 test	−0.04	0.80	0.26	0.065			−0.61	<0.0001	−0.58	<0.0001
Corneal staining score	0.14	0.33	−0.44	0.001	−0.61	<0.0001			0.67	<0.0001
Conjunctival staining score	0.13	0.35	−0.17	0.24	−0.58	<0.0001	0.67	<0.0001		

PD: Pain degree; FBUT: fluorescein break up time; R: Spearman’s rank correlation coefficient. Underline is added when the absolute value of R is 0.4 or more.

**Table 4:** Multivariate analysis of objective findings for pain degree by the least squares method.

	Multivariate Analysis 1		Multivariate Analysis 2		Multivariate Analysis 3		Multivariate Analysis 4	
Variables	Logarithmic Value	*p* Value	Logarithmic Value	*p* Value	Logarithmic Value	*p* Value	Logarithmic Value	*p* Value
Subtype classification of DE	1.534	0.029	1.635	0.023	1.467	0.034	1.340	0.046
Age	0.253	0.56	0.179	0.66	0.131	0.74	0.094	0.80
Sex	1.270	0.053	1.293	0.051	1.067	0.086	1.146	0.071
FBUT	0.608	0.25	0.485	0.33				
Schirmer 1 test	0.083	0.83			0.108	0.78		
Corneal staining score	0.323	0.48					0.016	0.96
Conjunctival staining score	0.132	0.74						

DE: dry eye; FBUT: fluorescein break up time. Underline is added when *p* value is less than 0.05. Underline is added when the absolute value of c is 1.300 or more.

## Supplementary Materials

The following are available online at https://www.mdpi.com/2075-4418/11/2/166/s1, Figure S1: Scatterplots of relationship between DE-related symptoms evaluated by DSQ-VAS and each pain subscale or total pain evaluated by SF-MPQ-2.
