# Supplementary material for: Evaluation of Eye-Pain Severity between Dry-Eye Subtypes
Source: Diagnostics (Basel). 2021 Jan 25;11(2):166. doi: 10.3390/diagnostics11020166 (PMC7911415; doi:10.3390/diagnostics11020166)
Supplement: Supplementary file 1 [file diagnostics-11-00166-s001.pdf]

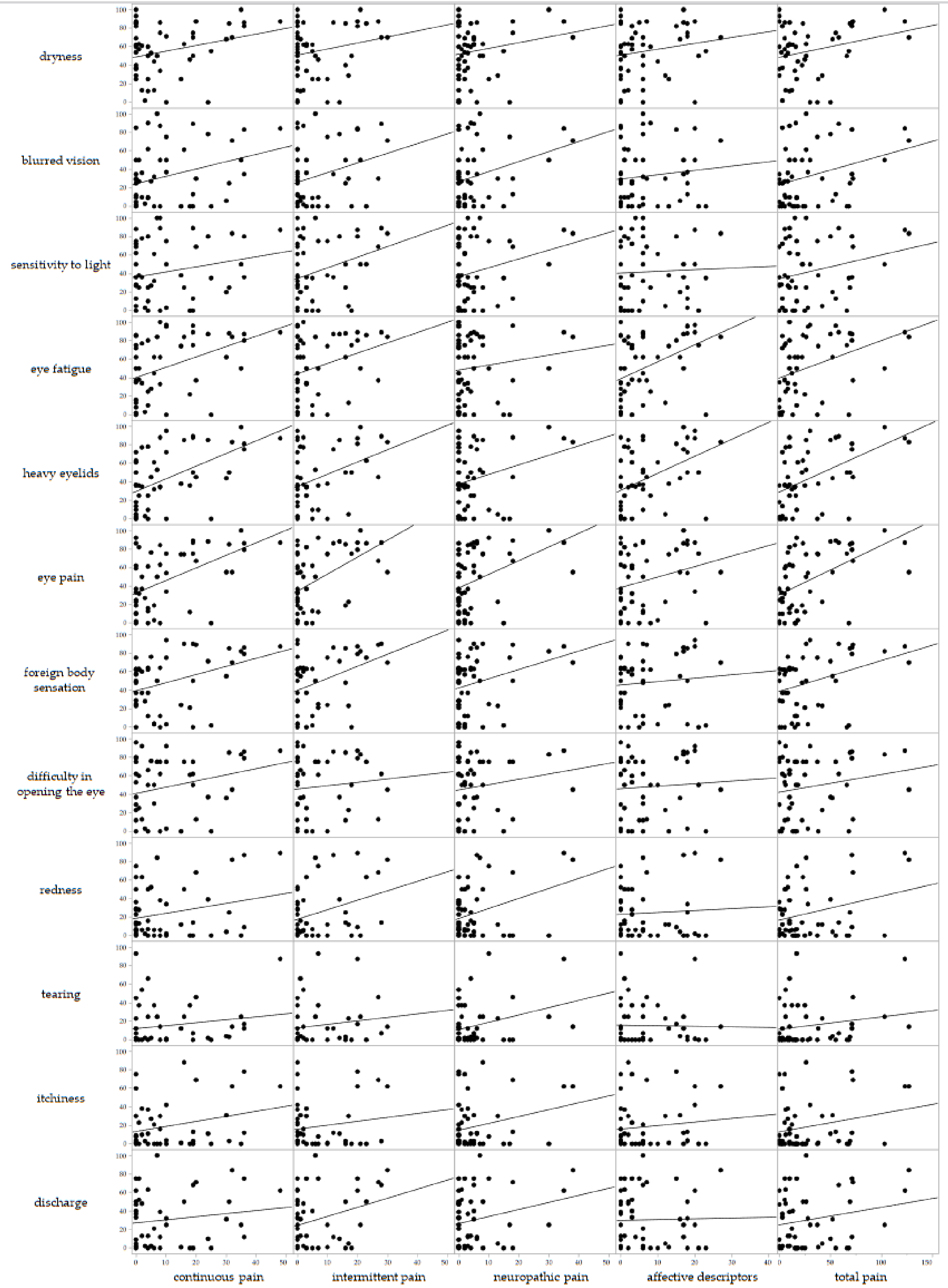

Figure S1. Scatterplots of relationship between DE-related symptoms evaluated by DSQ-VAS and each pain subscale or total pain evaluated by SF-MPQ-2.
